# Supplementary material for: Extracting multi-way chromatin contacts from Hi-C data
Source: PLoS Comput Biol. 2021 Dec 6;17(12):e1009669. doi: 10.1371/journal.pcbi.1009669 (PMC8675768; doi:10.1371/journal.pcbi.1009669)
Supplement: S1 Appendix — Table A. Genomic regions simulated in this work. Table B. Pearson correlation (PC), stratum-adjusted correlation (SCC8) and distance-corrected Pearson correlation (DCPC9) of the contact probabilities predicted by SBS and HLM compared with Capture-C1 and Tri-C2 experiments. Table C. Stratum adjusted correlation (SCC) and PC coefficients between Hi-C and HLM model. (PDF) [file pcbi.1009669.s001.pdf]

Supporting Information: Extracting multi-way chromatin contacts from Hi-C data

Lei Liu and Bokai Zhang
Key Laboratory of Optical Field Manipulation of Zhejiang Province, Department of Physics, Zhejiang Sci-Tech University, Hangzhou, China

Changbong Hyeon
Korea Institute for Advanced Study, Seoul 02455, South Korea

S1 APPENDIX

Derivation of n-body contact probability based on the cross-linking probability modeled with the Heaviside step function.

Pairwise contact probability

Along with Eq. 9, the pairwise contact probability assuming $F_1(r)$ can be determined by [1]

$$\begin{aligned} p^{(1)}_{ij} &= \int_0^\infty P(r_{ij})\Theta(r_c - r_{ij})dr_{ij} = \int_0^{r_c} P(r_{ij})dr_{ij} \\ &= \text{erf}(\gamma_{ij}^{1/2} r_c) - 2r_c\sqrt{\frac{\gamma_{ij}}{\pi}}e^{-\gamma_{ij}r_c^2}, \end{aligned} \tag{S1}$$

with $\text{erf}(x) = \frac{2}{\sqrt{\pi}}\int_0^x dte^{-t^2}$.

Three-body contact probability

The simultaneous contact probability among three sites i, j, and k assuming F_1, $p^{(1)}_{ijk}$, can be defined as

$$\begin{aligned} p^{(1)}_{ijk} &\equiv p\left((r_{ij} \leq r_c) \wedge (r_{ik} \leq r_c) \wedge (r_{jk} \leq r_c)\right) \\ &= \int_0^{r_c} p\left((r_{ik} \leq r_c) \wedge (r_{jk} \leq r_c) \mid r_{ij}\right) P(r_{ij})dr_{ij}, \end{aligned} \tag{S2}$$

where $p\left((r_{ik} \leq r_c) \wedge (r_{jk} \leq r_c) \mid r_{ij}\right)$ $(\equiv p^{(1)}_{ik,jk|r_{ij}})$ stands for the probability of the k-th monomer being simultaneously in contact with other two monomers conditioned with the distance r_{ij}, which we evaluated using cylindrical coordinates (ρ, ϕ, z). Under the conditions of $\vec{r}_i = (0, 0, r_{ij}/2)$ and $\vec{r}_j = (0, 0, -r_{ij}/2)$, the position of k-th monomer is still described in terms of the Gaussian distribution

$$P(\vec{r}_k) = Ce^{-\gamma_{ij,k}(z_k - z^*_{ij,k})^2}e^{-\gamma_{ij,k}\rho_k^2}, \tag{S3}$$

with the variance of the distribution $\gamma_{ij,k} = \det(\mathbf{K}_{ij})/\det(\mathbf{K}_{ijk})$, and a normalization constant $C = (\pi/\gamma_{ij,k})^{-3/2}$. $P(\vec{r}_k)$ is shifted along z-axis by

$$z^*_{ij,k} = \frac{r_{ij}}{2}\{(\mathbf{K}_{ij})^{-1} \cdot (\mathbf{k}^j_{\setminus\{i,j\}} - \mathbf{k}^i_{\setminus\{i,j\}})\}_k, \tag{S4}$$

where $\mathbf{k}^m_{\setminus\{i,j\}}$ is the m-th column of the matrix $\mathbf{K}$ after removing the i-th and the j-th rows. For $r_{ij} \leq r_c$, the conditional probability $q_{ik,jk|r_{ij}}$ equals to an integral over the intersection formed between two spheres of a radius r_c ,

$$\begin{aligned} p^{(1)}_{ik,jk|r_{ij}} &= \int_0^{2\pi} d\phi \int_{-D}^{D} dz \int_0^{R(z)} d\rho P(\vec{r}_k)\rho \\ &= C\int_0^{2\pi} d\phi \int_{-D}^{D} dz e^{-\gamma_{ij,k}(z - z^*_{ij,k})^2} \int_0^{R(z)} d\rho e^{-\gamma_{ij,k}\rho^2}\rho \\ &= -\sqrt{\frac{\gamma_{ij,k}}{\pi}}\int_{-D}^{D} dz e^{-\gamma_{ij,k}(z - z^*_{ij,k})^2}e^{-\gamma_{ij,k}\rho^2}\Bigg|_{\rho=0}^{\rho=R(z)} \\ &= \sqrt{\frac{\gamma_{ij,k}}{\pi}}\Big[\int_{-D}^{D} dz e^{-\gamma_{ij,k}(z - z^*_{ij,k})^2} \\ &\quad - e^{-\gamma_{ij,k}r_c^2}\int_{-D}^{0} dz e^{-\gamma_{ij,k}(z - z^*_{ij,k})^2}e^{\gamma_{ij,k}(\frac{r_{ij}}{2} - z)^2} \\ &\quad - e^{-\gamma_{ij,k}r_c^2}\int_0^{D} dz e^{-\gamma_{ij,k}(z - z^*_{ij,k})^2}e^{\gamma_{ij,k}(\frac{r_{ij}}{2} + z)^2}\Big] \\ &= \sqrt{\frac{\gamma_{ij,k}}{\pi}}\Big[I_0 + e^{-\gamma_{ij,k}r_c^2}I_- + e^{-\gamma_{ij,k}r_c^2}I_+\Big], \end{aligned} \tag{S5}$$

where $R(z) = (r_c^2 - (\frac{r_{ij}}{2} + |z|)^2)^{1/2}$ has been inserted in the third row. Considering that $D = r_c - r_{ij}/2$ (> 0), it is straightforward to calculate the integrals in the second to the last row of Eq. S5. One gets

$$I_0 = \begin{cases} \frac{1}{2}\sqrt{\frac{\pi}{\gamma_{ij,k}}}\big[\text{erf}(|z_-|) + \text{erf}(|z_+|)\big], & z_-z_+ < 0 \\ \frac{1}{2}\sqrt{\frac{\pi}{\gamma_{ij,k}}}\big|\text{erf}(|z_-|) - \text{erf}(|z_+|)\big|, & z_-z_+ \geq 0 \end{cases}, \tag{S6}$$

with $z_\mp = (\mp D - z^*_{ij,k})\gamma_{ij,k}^{1/2}$, and

$$I_\mp = \begin{cases} -D, & r_\mp = 0 \\ \frac{e^{(\gamma_{ij,k}r_+r_-)/4}}{\gamma_{ij,k}r_\mp}(1 - e^{\gamma_{ij,k}Dr_\mp}), & r_\mp \neq 0 \end{cases}, \tag{S7}$$

where $r_\mp = r_{ij} \pm 2z^*_{ij,k}$. The triplet contact probability in Eq. S2 can be computed numerically as a one-dimensional finite integral of a function of variable r_{ij}, together with Eqs. 9, S5-S7.

Conditional pairwise contact probability

Similarly to the discussions in the **Subsection: Comparison with SPRITE** in the main text, the first conditional pairwise contact probability, $p(r_{jk} \leq r_c \mid r_{ij} \leq r_c)$, can be calculated based on the relation that $p(r_{jk} \leq r_c \mid r_{ij} \leq r_c) = p^{(1)}_{jk|r_{ij}\leq r_c} = p^{(1)}_{ij,jk}/p^{(1)}_{ij}$, where

$$p^{(1)}_{ij,jk} = p((r_{jk} \leq r_c) \wedge (r_{ij} \leq r_c)) = \int_0^{r_c} P(r_{ij}) p(r_{jk} \leq r_c \mid r_{ij}) dr_{ij}. \quad \text{(S8)}$$

By setting $\vec{r}_i = (0, 0, r_{ij})$ and $\vec{r}_j = (0, 0, 0)$, we calculated $p(r_{jk} \leq r_c \mid r_{ij})$ ($\equiv p^{(1)}_{jk|r_{ij}}$), via a similar procedure of obtaining $p^{(1)}_{ik,jk|r_{ij}}$ (Eq.S5). The distribution of the k-th monomer, $P(\vec{r}_k)$, has the same form as Eq. S3, with the center being determined at $z^*_{ij,k} = r_{ij}\{(\mathbf{K}_{ij})^{-1} \cdot (-\mathbf{k}^i_{\setminus\{i,j\}})\}_k$. $p^{(1)}_{jk|r_{ij}}$ is evaluated by integrating over a sphere centered at the origin with a radius r_c,

$$\begin{aligned} p^{(1)}_{jk|r_{ij}} &= \int_0^{2\pi} d\phi \int_{-r_c}^{r_c} dz \int_0^{R(z)} d\rho P(\vec{r}_k)\rho \\ &= \sqrt{\frac{\gamma_{ij,k}}{\pi}} \Big[\int_{-r_c}^{r_c} dz e^{-\gamma_{ij,k}(z-z^*_{ij,k})^2} \\ &- \int_{-r_c}^{r_c} dz e^{-\gamma_{ij,k}(z-z^*_{ij,k})^2} e^{-\gamma_{ij,k}(r_c^2-z^2)} \Big] \\ &= \sqrt{\frac{\gamma_{ij,k}}{\pi}} \Big[I_0 - I_1 \Big], \end{aligned} \quad \text{(S9)}$$

where $R(z) = (r_c^2 - z^2)^{1/2}$, I_0 is given in Eq. S6 with $z_\mp = (\mp r_c - z^*_{ij,k})\gamma^{1/2}_{ij,k}$, and

$$I_1 = \begin{cases} 2r_c e^{-\gamma_{ij,k}(z^{*2}_{ij,k}+r_c^2)}, & z^*_{ij,k} = 0 \\ \frac{e^{-\gamma_{ij,k}(z^{*2}_{ij,k}+r_c^2)}}{\gamma_{ij,k} z^*_{ij,k}} \sinh(2\gamma_{ij,k} z^*_{ij,k} r_c), & z^*_{ij,k} \neq 0 \end{cases}. \quad \text{(S10)}$$

After computing $p^{(1)}_{ij,jk}$ with Eq. S8-S9, the second conditional pairwise contact probability can be determined by

$$p^{(1)}_{jk|r_{ij}>r_c} = \frac{p^{(1)}_{jk} - p^{(1)}_{ij,jk}}{1 - p^{(1)}_{ij}}, \quad \text{(S11)}$$

where the marginal contact probabilities $p^{(1)}_{ij}$ and $p^{(1)}_{jk}$ are given by Eq. S1 in this case.

Note that neither $p^{(1)}_{jk|r_{ij}\leq r_c}$ nor $p^{(1)}_{ij,jk}$ equals to $p^{(1)}_{ijk}$ if $r_c > 0$. Although both loci are concurrently in contact with the j-th site, the contact between the i-th and k-th loci is not guaranteed. To be precise, what Bintu *et al.* have quantified [2] are not higher-order chromatin contacts.

TABLE A. Genomic regions simulated in this work.

Species	Chr	Region [a]	Res[b]	N	Cell line	2-body[c]	PC [d]	Viewpoint	3-body[e]	PC [f]	Figure
mouse	11	32,000,000-32,300,000	2	150	ES	[3]	0.99	32,151,060-32,151,883	[4]	0.80	Fig 3C
								32,137,176-32,137,426	[4]	0.89	Fig 3D
					ERY	[3]	0.98	32,151,060-32,151,883	[4]	0.75	Fig 3C
								32,137,176-32,137,426	[4]	0.85	Fig 3D
	8	120,800,000-122,080,000	10	128	HAP1 WT	[5]	0.97	121,126,666-121,145,791	[6]	0.83	Fig 4B
								121,941,808-121,960,933	[6]	0.71	Fig 4C
					HAP1 ΔWAPL	[5]	0.96	121,126,666-121,145,791	[6]	0.81	Fig 4B
								121,941,808-121,960,933	[6]	0.73	Fig 4C
	7	110,836,514-111,140,514	8	38	ES	[7]	1.00	110,955,508-110,955,666	[4]	0.62	S10B Fig
								111,009,550-111,009,749	[4]	0.88	S10B Fig
	18	37,059,654-37,399,654	5	68	NPC	[7]	0.98	37,109,610-37,114,710	[6]	0.84	S11B Fig
								37,167,503-37,172,603	[6]	0.76	S11B Fig
								37,247,128-37,252,228	[6]	0.76	S11B Fig
								37,335,329-37,340,429	[6]	0.88	S11B Fig
								37,374,676-37,379,776	[6]	0.70	S11B Fig
human	18	60,675,000-61,120,000	5	89	GM12878	[8]	0.99	60,980,000-60,990,000	[9]	0.18	Fig 5C
	12	11,690,000-12,210,000	5	104	GM12878	[8]	0.99	11,800,000-11,815,000	[9]	0.16	S12C Fig
	21	29,370,000-30,600,000	30	41	IMR90	[8]	0.99				S13 Fig

[a] The reference genome assemblies of mouse and human are mm9 and hg19, respectively.
[b] The model resolutions in units of kb.
[c] References of the experimental pairwise contacts datasets.
[d] Pearson correlation coefficient of two-body contact probabilities between Hi-C/Capture-C and HLM.
[e] References of the experimental triplet contacts datasets.
[f] Pearson correlation coefficient of three-body contact probabilities between 3-body experiments and HLM.

TABLE B. Pearson correlation (PC), stratum-adjusted correlation (SCC [10]) and distance-corrected Pearson correlation (DCPC [11]) of the contact probabilities predicted by SBS [12] and HLM compared with Capture-C [3] and Tri-C [4] experiments ("NA" stands for not available).

		Pairwise contacts		Triplet contacts at R2		Triplet contacts at HS-39	
		(SBS, Cap-C)	(HLM, Cap-C)	(SBS, Tri-C)	(HLM, Tri-C)	(SBS, Tri-C)	(HLM, Tri-C)
ES	PC	0.96	0.99	0.80	0.80	0.84	0.89
	SCC	0.75	0.88	0.66	0.72	0.77	0.82
	DCPC	0.87	0.91	NA	0.68	NA	0.65
ERY	PC	0.96	0.98	0.80	0.75	0.77	0.85
	SCC	0.92	0.93	0.48	0.66	0.79	0.80
	DCPC	0.91	0.97	NA	0.70	NA	0.72

TABLE C. Stratum adjusted correlation (SCC) and PC coefficients between Hi-C and HLM model, which was trained with different cross-linking probabilities, cost functions, and optimizers (see also S12 Fig). Each coefficient value is the averaged result of five independent trainings.

	RS	GD	RMS	ADAM
SCC F_0 L_0	-1.872e-02	5.684e-02	7.326e-01	7.250e-01
L_1	7.700e-02	7.400e-01	7.936e-01	**8.418e-01**
L_2	7.191e-01	5.811e-01	8.159e-01	**8.537e-01**
L_3	-1.872e-02	9.641e-02	7.960e-01	8.390e-01
PC F_0 L_0	8.194e-01	9.378e-01	9.829e-01	9.835e-01
L_1	8.731e-01	9.818e-01	9.884e-01	**9.914e-01**
L_2	9.836e-01	9.753e-01	9.901e-01	**9.924e-01**
L_3	8.1934-01	9.025e-01	9.883e-01	9.913e-01
SCC F_1 L_0	6.318e-02	6.798e-02	7.661e-01	8.073e-01
L_1	3.944e-01	7.488e-01	7.933e-01	8.441e-01
L_2	7.161e-01	5.809e-01	8.155e-01	**8.543e-01**
L_3	8.207e-02	1.040e-01	7.956e-01	**8.454e-01**
PC F_1 L_0	8.561e-01	9.339e-01	9.843e-01	9.900e-01
L_1	9.423e-01	9.825e-01	9.884e-01	9.917e-01
L_2	9.834e-01	9.753e-01	9.901e-01	**9.924e-01**
L_3	8.627e-01	9.003e-01	9.885e-01	**9.918e-01**

[1] Liu L, Kim MH, Hyeon C. Heterogeneous loop model to infer 3D chromosome structures from Hi-C. Biophys J. 2019;117(3):613–625. doi:10.1016/j.bpj.2019.06.032.

[2] Bintu B, Mateo LJ, Su JH, Sinnott-Armstrong NA, Parker M, Kinrot S, et al. Super-resolution chromatin tracing reveals domains and cooperative interactions in single cells. Science. 2018;362(6413):eaau1783. doi:10.1126/science.aau1783.

[3] Oudelaar AM, Beagrie RA, Gosden M, de Ornellas S, Georgiades E, Kerry J, et al. Dynamics of the 4D genome during in vivo lineage specification and differentiation. Nat Comm. 2020;11(1):2722. doi:10.1038/s41467-020-16598-7.

[4] Oudelaar AM, Davies JOJ, Hanssen LLP, Telenius JM, Schwessinger R, Liu Y, et al. Single-allele chromatin interactions identify regulatory hubs in dynamic compartmentalized domains. Nat Genet. 2018;50:1744–1751. doi:10.1038/s41588-018-0253-2.

[5] Haarhuis JHI, van der Weide RH, Blomen A Vincent, Yáñez-Cuna JO, Amendola M, van Ruiten MS, et al. The Cohesin Release Factor WAPL Restricts Chromatin Loop Extension. Cell. 2017;169(4):693–707. doi:10.1016/j.cell.2017.04.013.

[6] Allahyar A, Vermeulen C, Bouwman BAM, Krijger PHL, Verstegen MJAM, Geeven G, et al. Enhancer hubs and loop collisions identified from single-allele topologies. Nat Genet. 2018;50(8):1151–1160. doi:10.1038/s41588-018-0161-5.

[7] Bonev B, Cohen NM, Szabo Q, Fritsch L, Papadopoulos GL, Lubling Y, et al. Multiscale 3D Genome Rewiring during Mouse Neural Development. Cell. 2017;171(3):557 – 572.e24. doi:https://doi.org/10.1016/j.cell.2017.09.043.

[8] Rao SSP, Huntley MH, Durand NC, Stamenova EK, Bochkov ID, Robinson JT, et al. A 3D Map of the Human Genome at Kilobase Resolution Reveals Principles of Chromatin Looping. Cell. 2014;159(7):1665–1680. doi:10.1016/j.cell.2014.11.021.

[9] Quinodoz SA, Ollikainen N, Tabak B, Palla A, Schmidt JM, Detmar E, et al. Higher-Order Interchromosomal Hubs Shape 3D Genome Organization in the Nucleus. Cell. 2018;174(3):744 – 757.e24. doi:https://doi.org/10.1016/j.cell.2018.05.024.

[10] Yang T, Zhang F, Yardimci GG, Song F, Hardison RC, Noble WS, et al. HiCRep: assessing the reproducibility of Hi-C data using a stratum-adjusted correlation coefficient. Genome Res. 2017;27:1939–1949. doi:10.1101/gr.220640.117.

[11] Bianco S, Lupiáñez DG, Chiariello AM, Annunziatella C, Kraft K, Schöpflin R, et al. Polymer physics predicts the effects of structural variants on chromatin architecture. Nat Genetics. 2018;50:662–667.

[12] Chiariello AM, Bianco S, Oudelaar AM, Esposito A, Annunziatella C, Fiorillo L, et al. A Dynamic Folded Hairpin Conformation Is Associated with alpha-Globin Activation in Erythroid Cells. Cell Rep. 2020;30(7):2125–2135. doi:10.1016/j.celrep.2020.01.044.

[13] Liu L, Kim MH, Hyeon C. Heterogeneous loop model to infer 3D chromosome structures from Hi-C. Biophys J. 2019;117(3):613–625. doi:10.1016/j.bpj.2019.06.032.

[14] Bintu B, Mateo LJ, Su JH, Sinnott-Armstrong NA, Parker M, Kinrot S, et al. Super-resolution chromatin tracing reveals domains and cooperative interactions in single cells. Science. 2018;362(6413):eaau1783. doi:10.1126/science.aau1783.

[15] Oudelaar AM, Beagrie RA, Gosden M, de Ornellas S, Georgiades E, Kerry J, et al. Dynamics of the 4D genome during in vivo lineage specification and differentiation. Nat Comm. 2020;11(1):2722. doi:10.1038/s41467-020-16598-7.

[16] Oudelaar AM, Davies JOJ, Hanssen LLP, Telenius JM, Schwessinger R, Liu Y, et al. Single-allele chromatin interactions identify regulatory hubs in dynamic compartmentalized domains. Nat Genet. 2018;50:1744–1751. doi:10.1038/s41588-018-0253-2.

[17] Haarhuis JHI, van der Weide RH, Blomen A Vincent, Yáñez-Cuna JO, Amendola M, van Ruiten MS, et al. The Cohesin Release Factor WAPL Restricts Chromatin Loop Extension. Cell. 2017;169(4):693–707. doi:10.1016/j.cell.2017.04.013.

[18] Allahyar A, Vermeulen C, Bouwman BAM, Krijger PHL, Verstegen MJAM, Geeven G, et al. Enhancer hubs and loop collisions identified from single-allele topologies. Nat Genet. 2018;50(8):1151–1160. doi:10.1038/s41588-018-0161-5.

[19] Bonev B, Cohen NM, Szabo Q, Fritsch L, Papadopoulos GL, Lubling Y, et al. Multiscale 3D Genome Rewiring during Mouse Neural Development. Cell. 2017;171(3):557 – 572.e24. doi:https://doi.org/10.1016/j.cell.2017.09.043.

[20] Rao SSP, Huntley MH, Durand NC, Stamenova EK, Bochkov ID, Robinson JT, et al. A 3D Map of the Human Genome at Kilobase Resolution Reveals Principles of Chromatin Looping. Cell. 2014;159(7):1665–1680. doi:10.1016/j.cell.2014.11.021.

[21] Quinodoz SA, Ollikainen N, Tabak B, Palla A, Schmidt JM, Detmar E, et al. Higher-Order Interchromosomal Hubs Shape 3D Genome Organization in the Nucleus. Cell. 2018;174(3):744 – 757.e24. doi:https://doi.org/10.1016/j.cell.2018.05.024.

[22] Yang T, Zhang F, Yardimci GG, Song F, Hardison RC, Noble WS, et al. HiCRep: assessing the reproducibility of Hi-C data using a stratum-adjusted correlation coefficient. Genome Res. 2017;27:1939–1949. doi:10.1101/gr.220640.117.

[23] Bianco S, Lupiáñez DG, Chiariello AM, Annunziatella C, Kraft K, Schöpflin R, et al. Polymer physics predicts the effects of structural variants on chromatin architecture. Nat Genetics. 2018;50:662–667.

[24] Chiariello AM, Bianco S, Oudelaar AM, Esposito A, Annunziatella C, Fiorillo L, et al. A Dynamic Folded Hairpin Conformation Is Associated with alpha-Globin Activation in Erythroid Cells. Cell Rep. 2020;30(7):2125–2135. doi:10.1016/j.celrep.2020.01.044.
