# Supplementary figures and images for: Extracting multi-way chromatin contacts from Hi-C data
Source: PLoS Comput Biol. 2021 Dec 6;17(12):e1009669. doi: 10.1371/journal.pcbi.1009669 (PMC8675768; doi:10.1371/journal.pcbi.1009669)

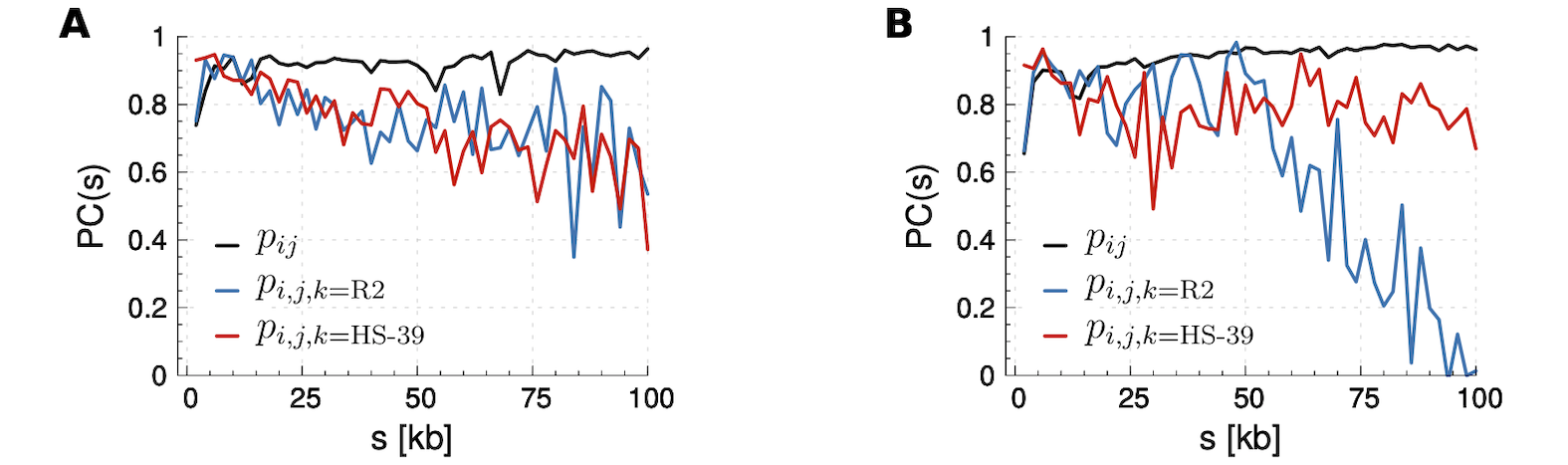

Supplement: S1 Fig — Stratified PC at α-globin locus of mouse (A) ES and (B) erythroid cells compared with Capture-C (2-body) and Tri-C (3-body) experiments. (TIF) [file pcbi.1009669.s002.tif]

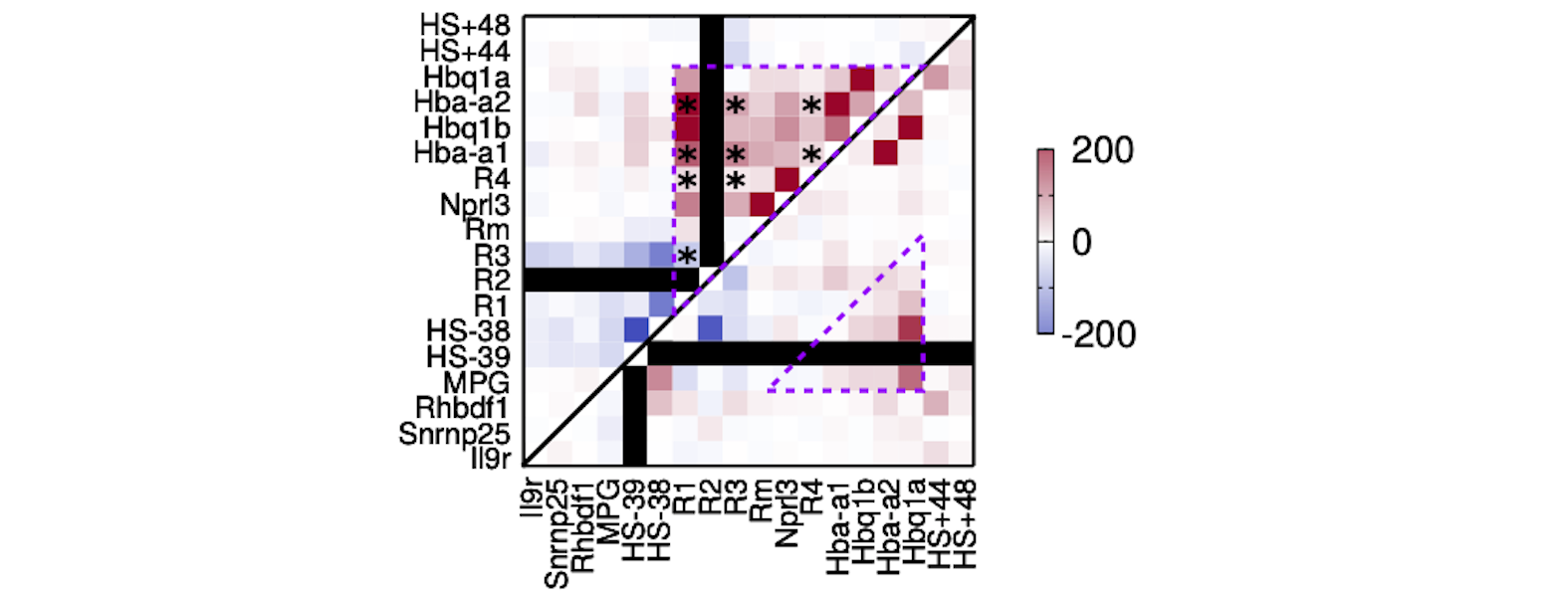

Supplement: S2 Fig — The analysis was done at the viewpoints of R2 (top) and HS-39 (bottom). Following the statistical analysis in the experiment [17], we use the symbol * to mark all triplet interactions with significant changes (P < 0.01). The erythroid cell-specific regulatory hub and diffuse interactions among CTCF boundary sites, which are highlighted by dashed triangles, are both captured by Tri-C and our theory (Fig 3E). (TIF) [file pcbi.1009669.s003.tif]

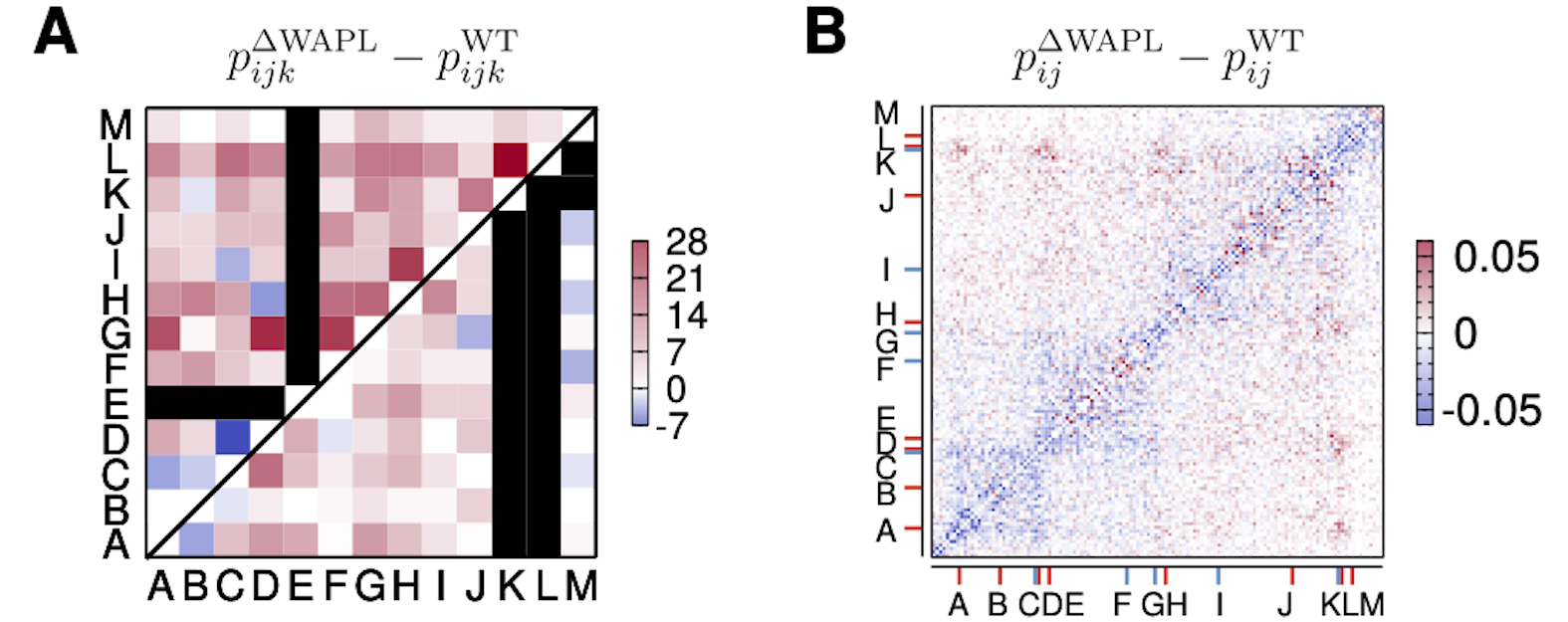

Supplement: S3 Fig — (A) The triplet contacts from MC-4C data [18] with respect to the viewpoints of E (top) and K (bottom). The triplet contacts predicted by HLM are shown in Fig 4D. (B) The enrichment of pairwise contacts between long-range CTCF binding sites (off-diagonal elements in red corresponding to (pijΔWAPL-pijWT)≳0.05) is counteracted by the depletion of contacts in the domains flanked by short-range CTCF binding sites (matrix elements along the diagonal block in blue corresponding to (pijΔWAPL-pijWT)≲-0.05). The data of pairwise contacts are obtained from Hi-C data [63]. (TIF) [file pcbi.1009669.s004.tif]

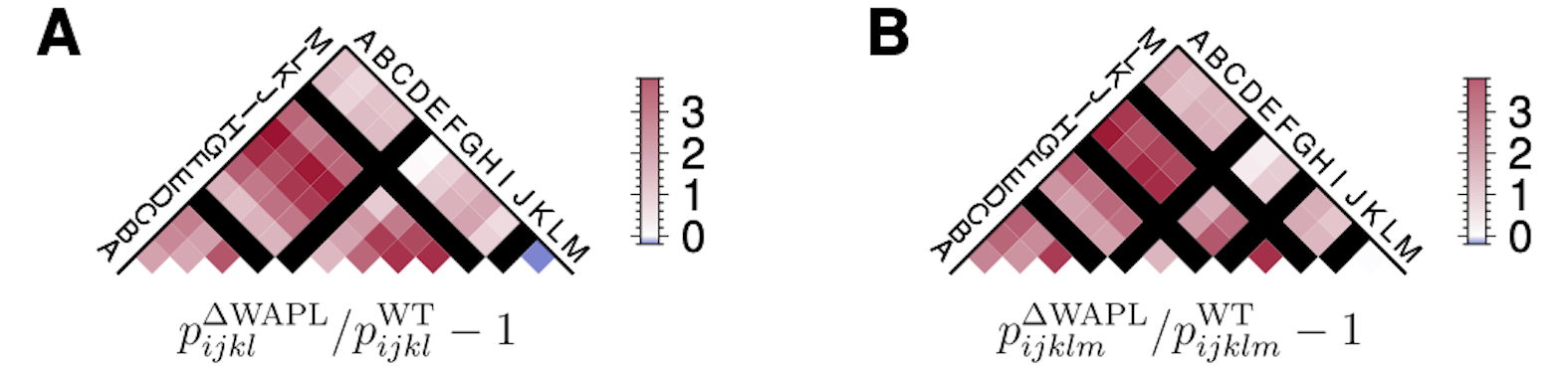

Supplement: S4 Fig — Fold changes of (A) four-body contacts double-anchored at sites E, K and (B) five-body contacts triple-anchored at sites E, H and K. The absolute change of contact probabilities is shown in Fig 4F and 4G, respectively. (TIF) [file pcbi.1009669.s005.tif]

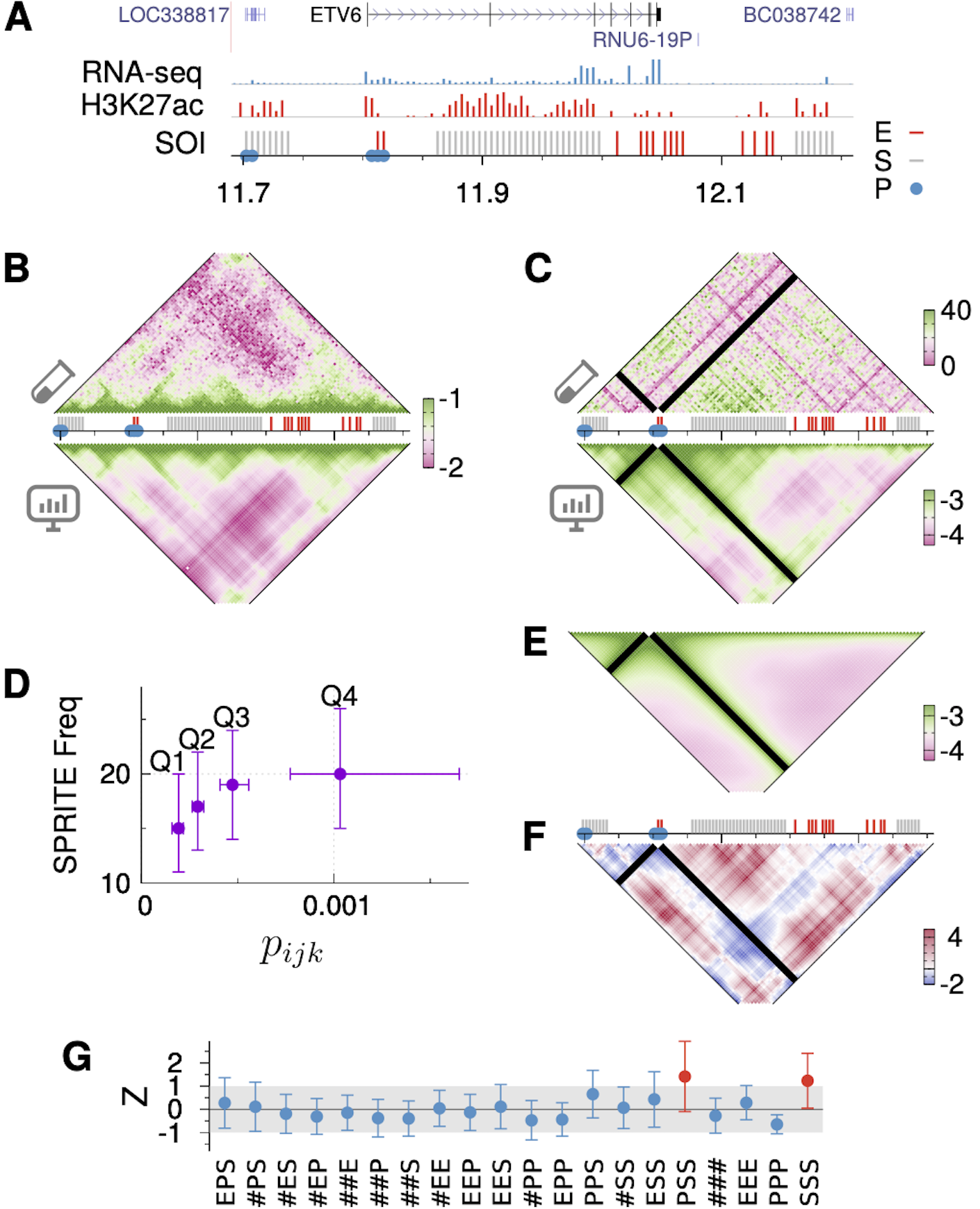

Supplement: S5 Fig — Comparison between HLM and SPRITE similarly to those of Fig 5 in a 0.52 Mb region on human chr12. (C) From the viewpoint of the promoter of active ETV6 gene which encodes an ETS family transcription factor. (TIF) [file pcbi.1009669.s006.tif]

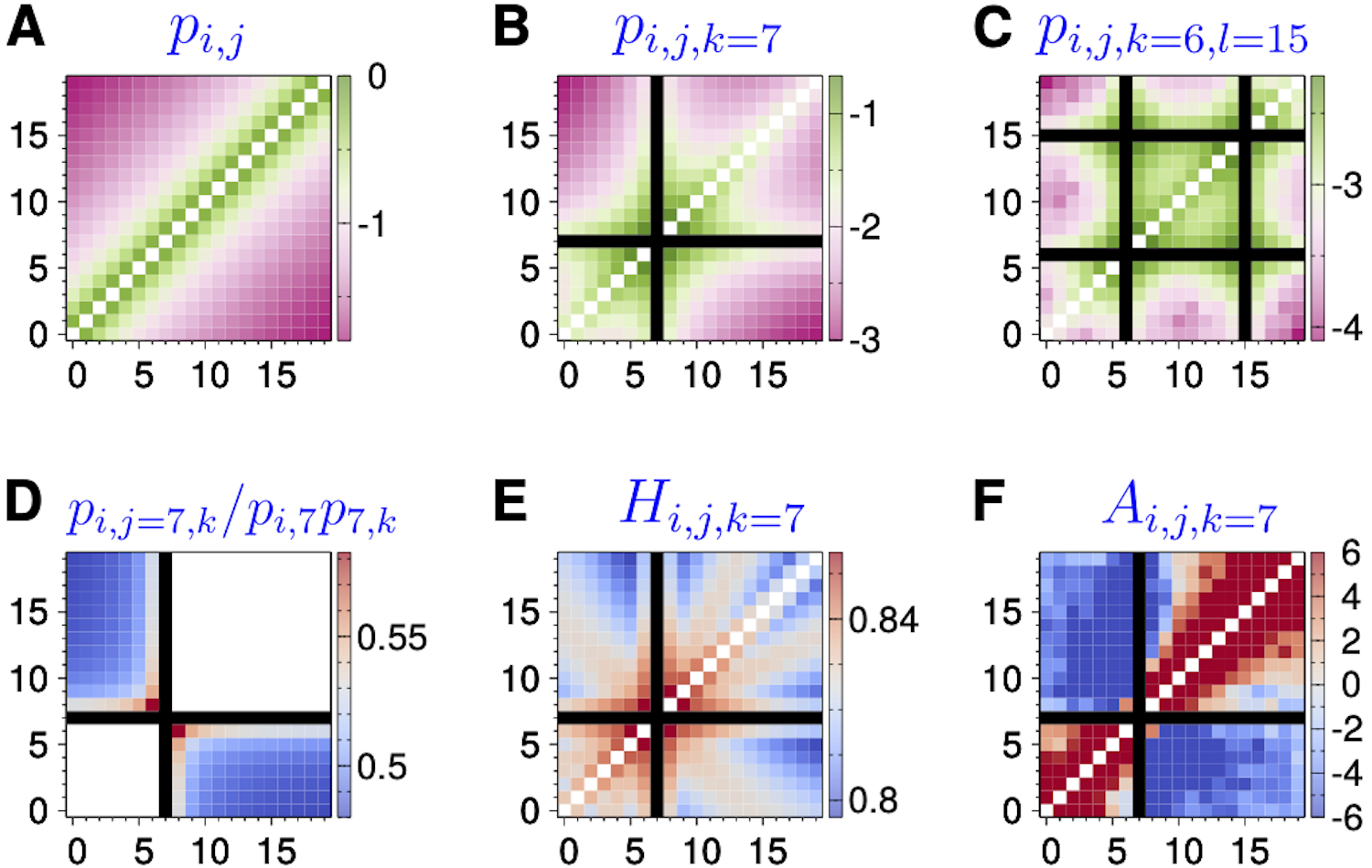

Supplement: S6 Fig — Same as Fig 6 but using F1(r) (Heaviside step function) as the cross-linking probability. (TIF) [file pcbi.1009669.s007.tif]

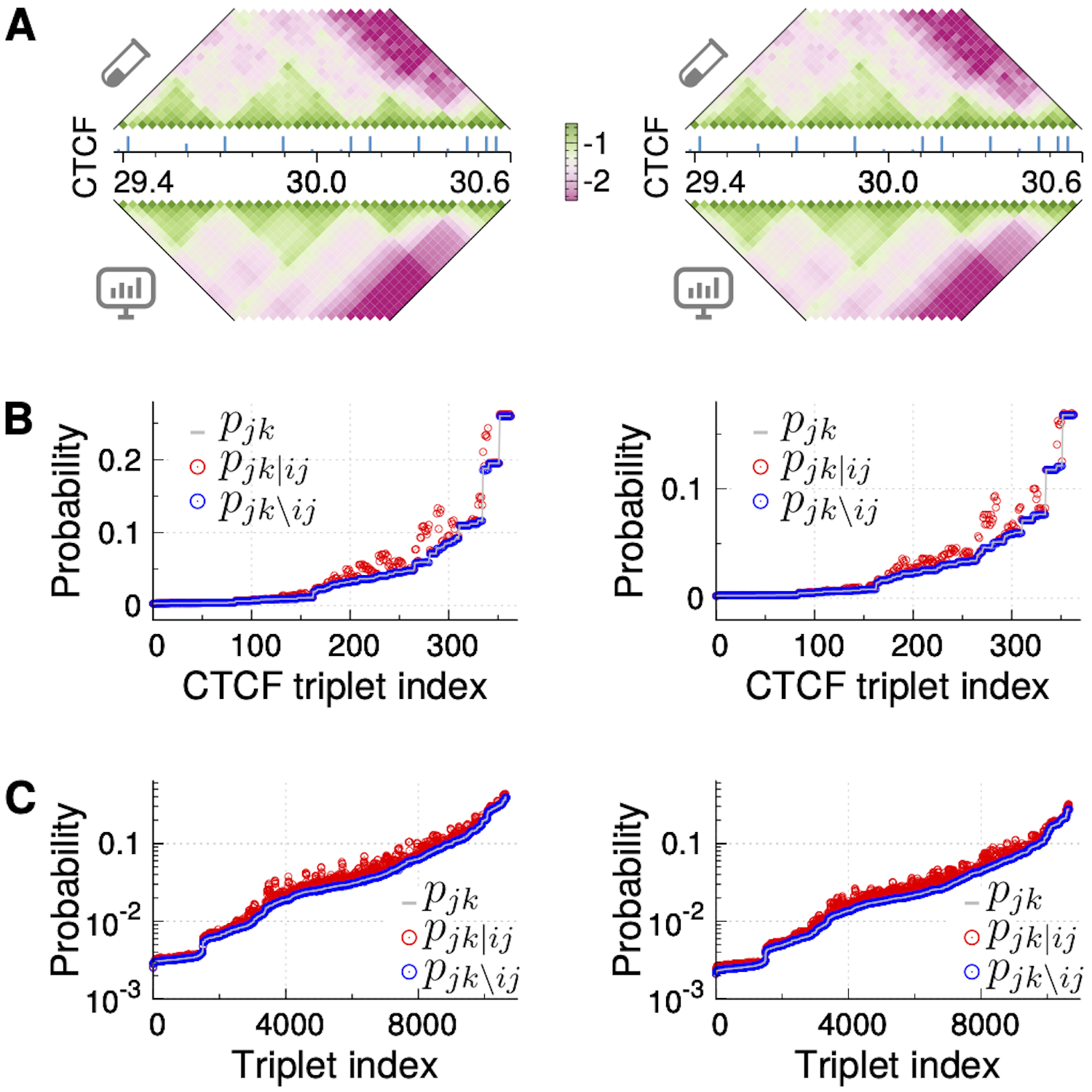

Supplement: S7 Fig — (A) Heatmaps of log10(pij) in a 1.23-Mb region on chr21 of IMR90 cells from Hi-C and log10(pij) from HLM. (B) Comparison between the unconditioned contact probability pjk, the conditional contact probability, pjk∣ij, and pjk\ij, calculated for CTCF-site triplets. (C) pjk, pjk∣ij, and pjk\ij calculated for all triplets ijk of i < j < k, which are sorted in an ascending order of pjk. (TIF) [file pcbi.1009669.s008.tif]

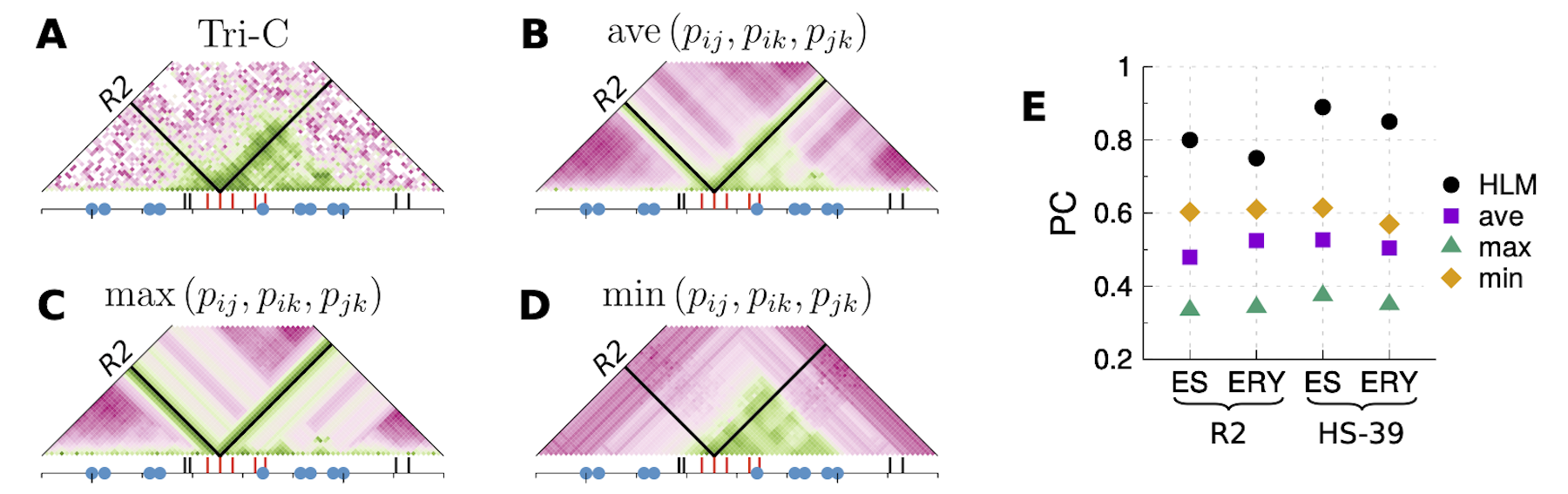

Supplement: S8 Fig — (A) Three-body contact matrix at α-globin locus of mouse erythroid cells measured by Tri-C experiment or predicted by using three simple rules (B-D). (E) Pearson correlations between the results from 4 methods and the experiment. (TIF) [file pcbi.1009669.s009.tif]

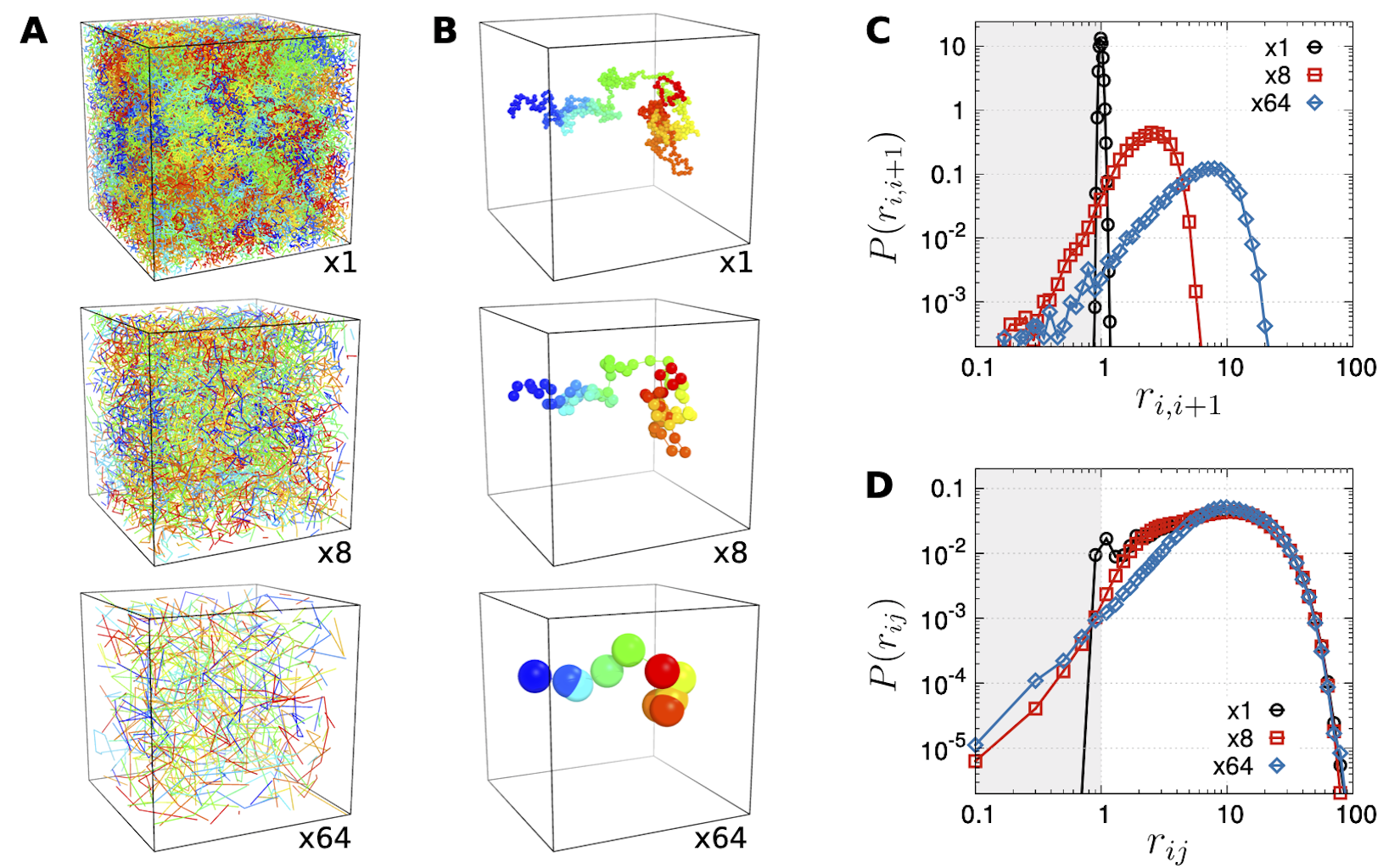

Supplement: S9 Fig — (A) A typical configuration of a dense polymer melt and (B) one polymer chain in the melt at three levels of coarse graining. The beads in (B) are colored differently along the chain, with a diameter of the most probable bond length at the corresponding scales. (C) Probability of the bond length and (D) intra-chain pairwise monomer distance. (TIF) [file pcbi.1009669.s010.tif]

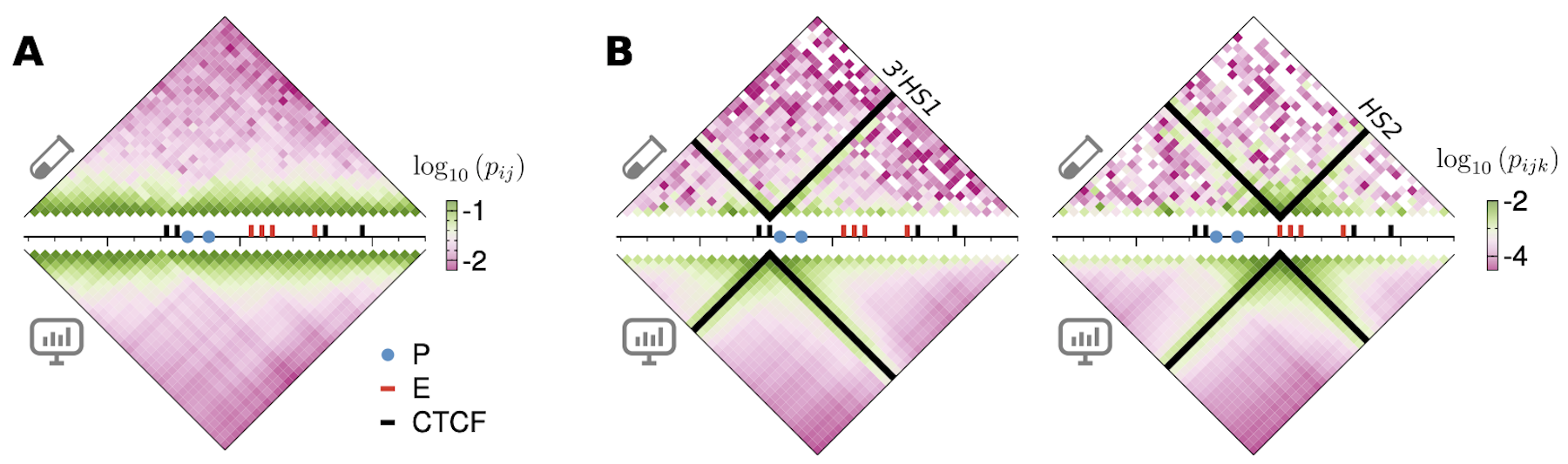

Supplement: S10 Fig — (A) Pairwise contact probability from Hi-C [70] compared with HLM, which has a PC of 0.996. (B) Triplet contact probabilities from Tri-C compared with HLM, which are anchored at 3’HS1 and HS2 with PCs of 0.62 and 0.88, respectively. (TIF) [file pcbi.1009669.s011.tif]

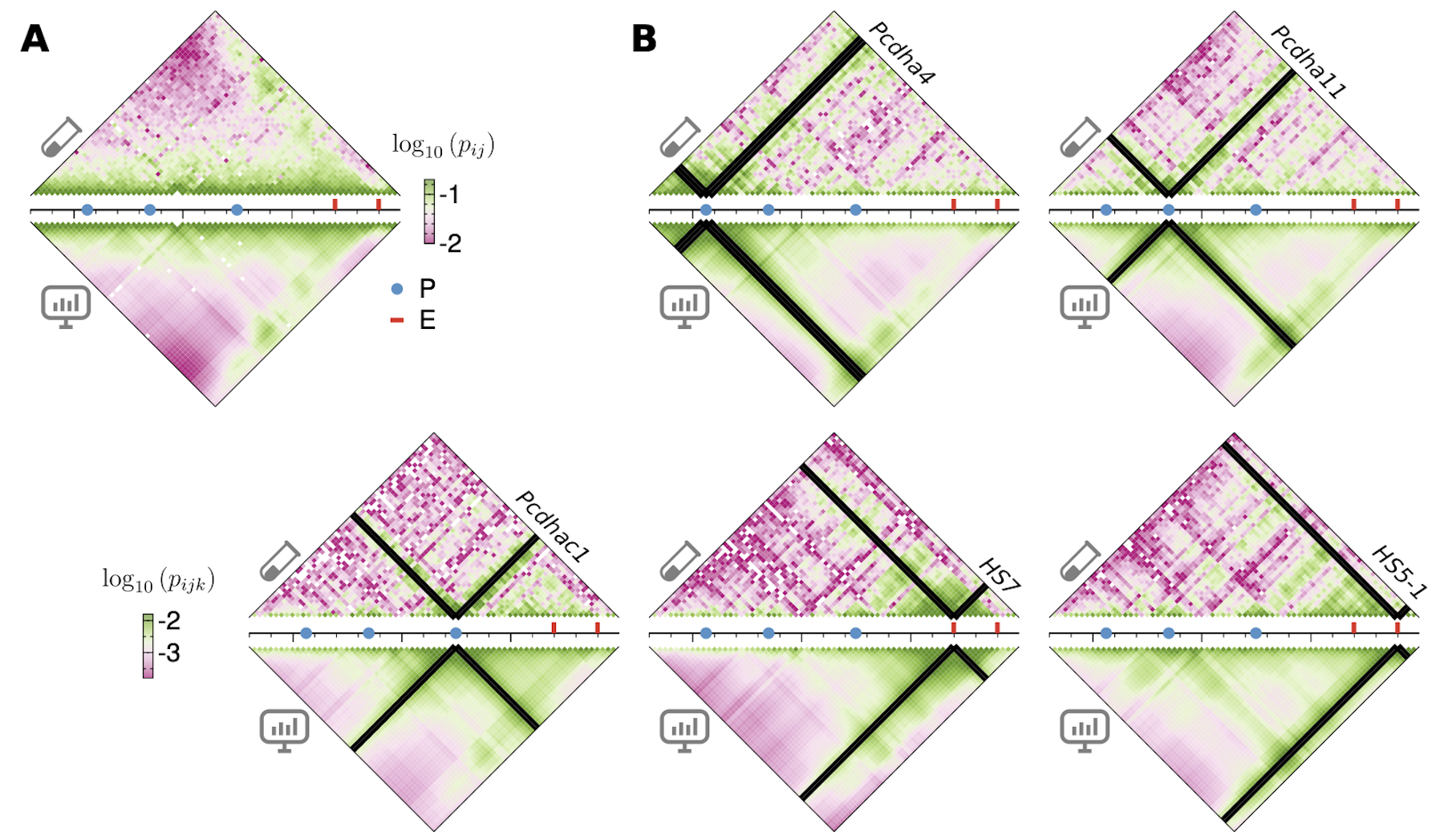

Supplement: S11 Fig — Compared with the MC-4C dataset, (A) the pairwise contact probability has a PC of 0.98, and (B) the triplet contact probabilities have PCs of 0.84, 0.76, 0.76, 0.88, and 0.70 at the viewpoint of Pcdhα1, Pcdhα11, Pcdhαc1, HS7, and HS5–1, respectively. (TIF) [file pcbi.1009669.s012.tif]

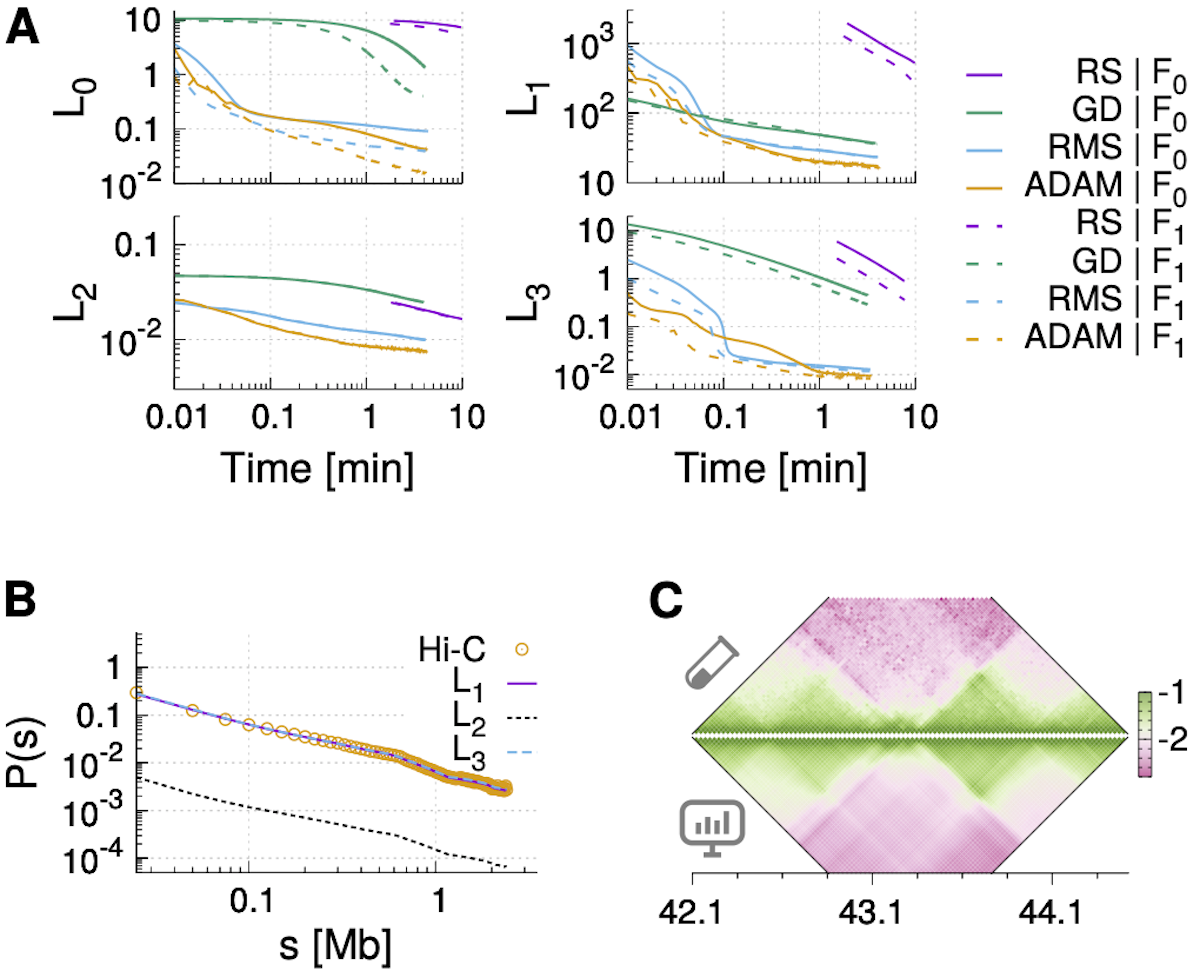

Supplement: S12 Fig — We trained a polymer model of a 2.4-Mb genomic region on chr8 in mouse ES cells at 25-kb resolution [70]. (A) The trajectories of various cost functions L in a log-log scale, minimized by using one of the four methods (RS, GD, RMSprop, and ADAM) with different cross-linking probabilities Fα (α = 0, 1). (B) Comparing P(s) from Hi-C and from three models, which were all trained with ADAM using F0, but with different forms of the cost functions. (C) Comparison of log10(pij) from Hi-C (top) with that from the model trained by minimizing L1 with ADAM (bottom). (TIF) [file pcbi.1009669.s013.tif]

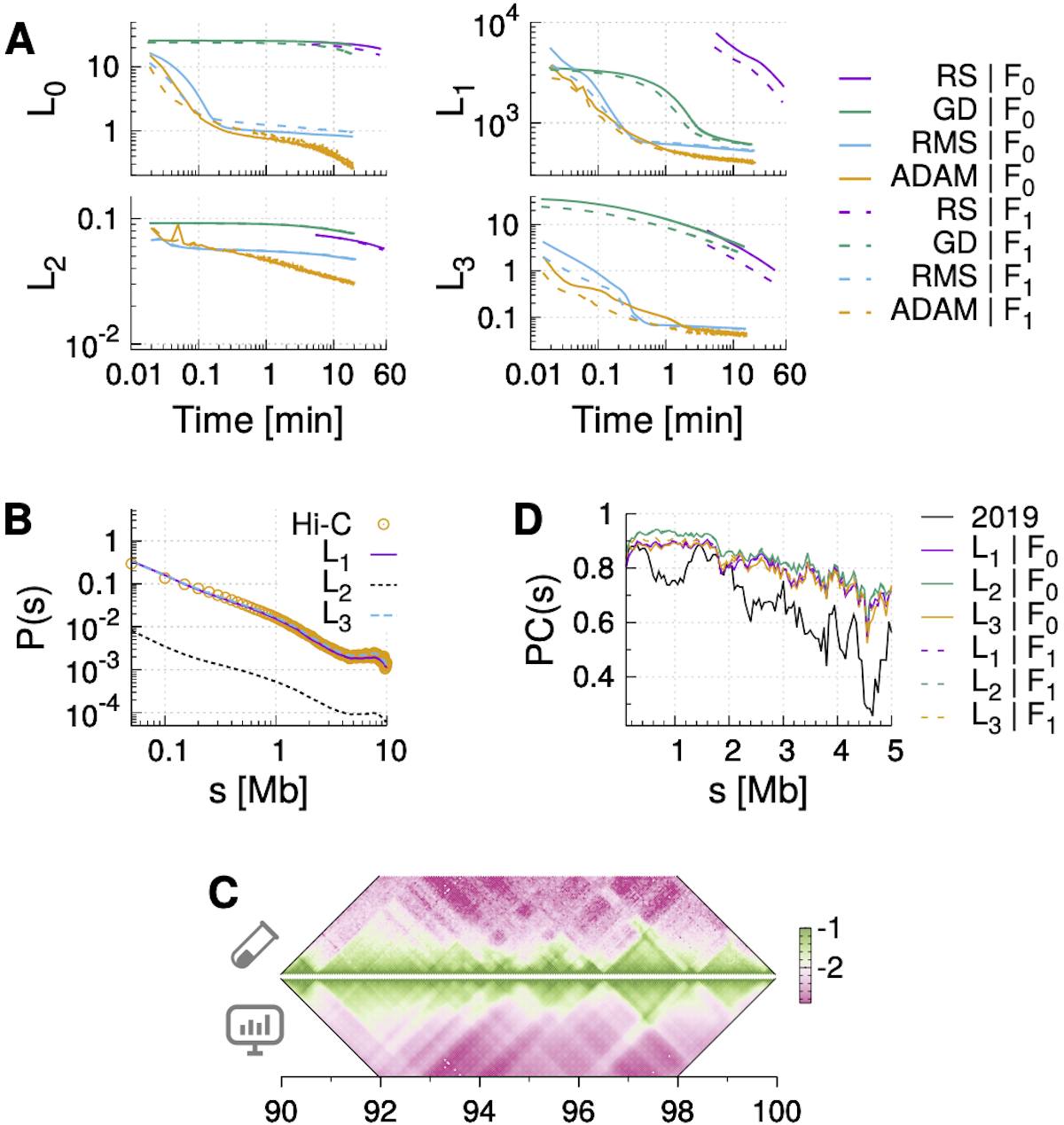

Supplement: S13 Fig — (A-C) Same as the caption of S12 Fig. (D) Pearson correlations between Hi-C and HLM in our previous work [50] (the black line), and new models trained in this work (the colored lines) as a function of genomic separation, s. (TIF) [file pcbi.1009669.s014.tif]
